# Supplementary material for: WntA expression and wing transcriptomics illuminate the evolution of stripe patterns in skipper butterflies
Source: Biol Open. 2025 Nov 26;14(11):bio062297. doi: 10.1242/bio.062297 (PMC12690525; doi:10.1242/bio.062297)
Supplement: Supplementary information [file biolopen-14-062297-s1.pdf]

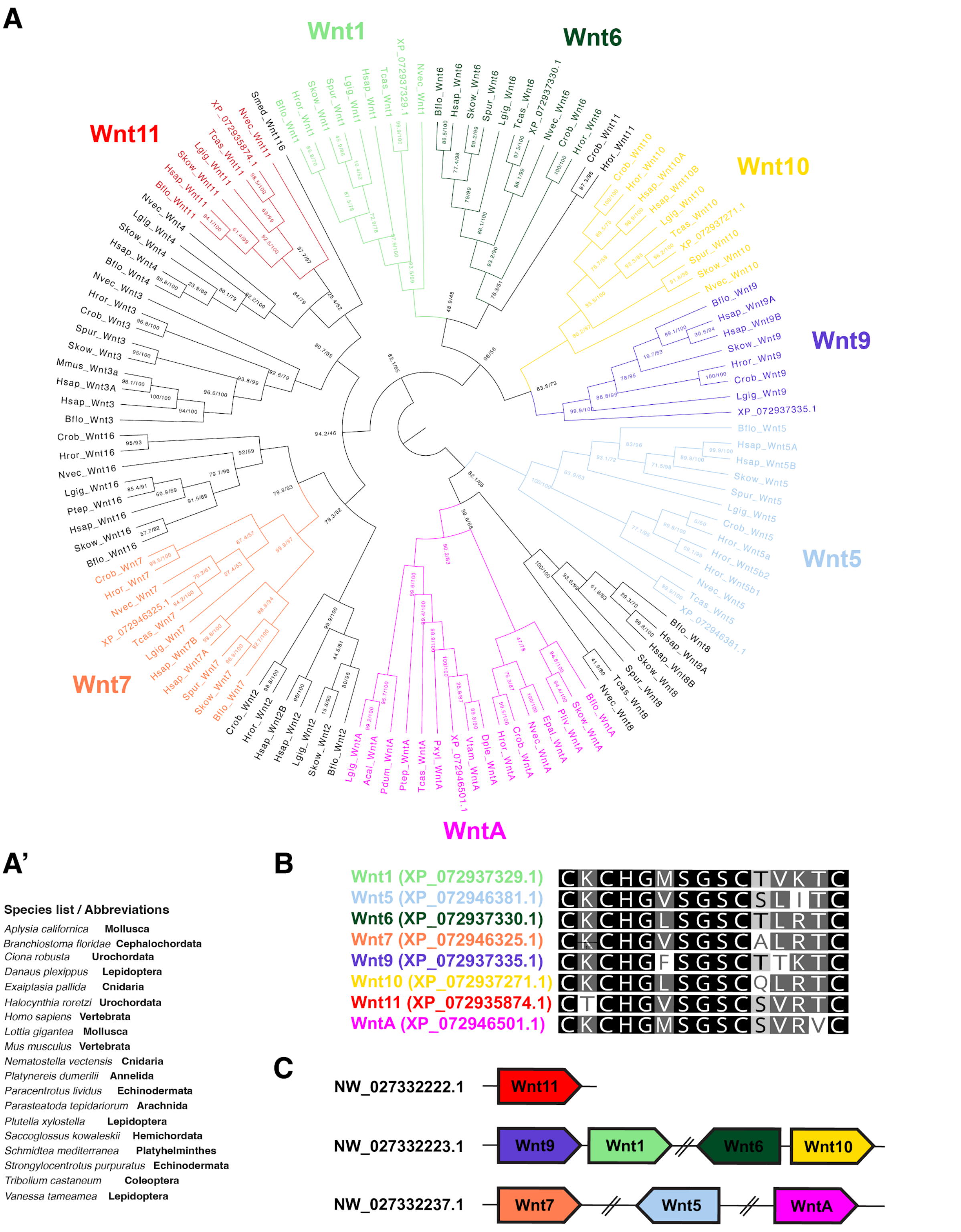

Fig. S1. Assignment of *E. clarus* Wnt ligand family genes to eight orthology groups. A. Maximum likelihood reconstruction of *E. clarus* and reference Wnt proteins (Hanly et al., 2021). Branch support is indicated by SH-aLRT % values / ultrafast bootstrap % values. **A’.** Species abbreviation key for the phylogeny. **B.** Amino-acid alignment of the eight *E. clarus* Wnt thumb regions. **C.** Arrangement of Wnt genes within the *E. clarus WU_Ecla_fem_2.2* genome assembly.

Biology Open • Supplementary information

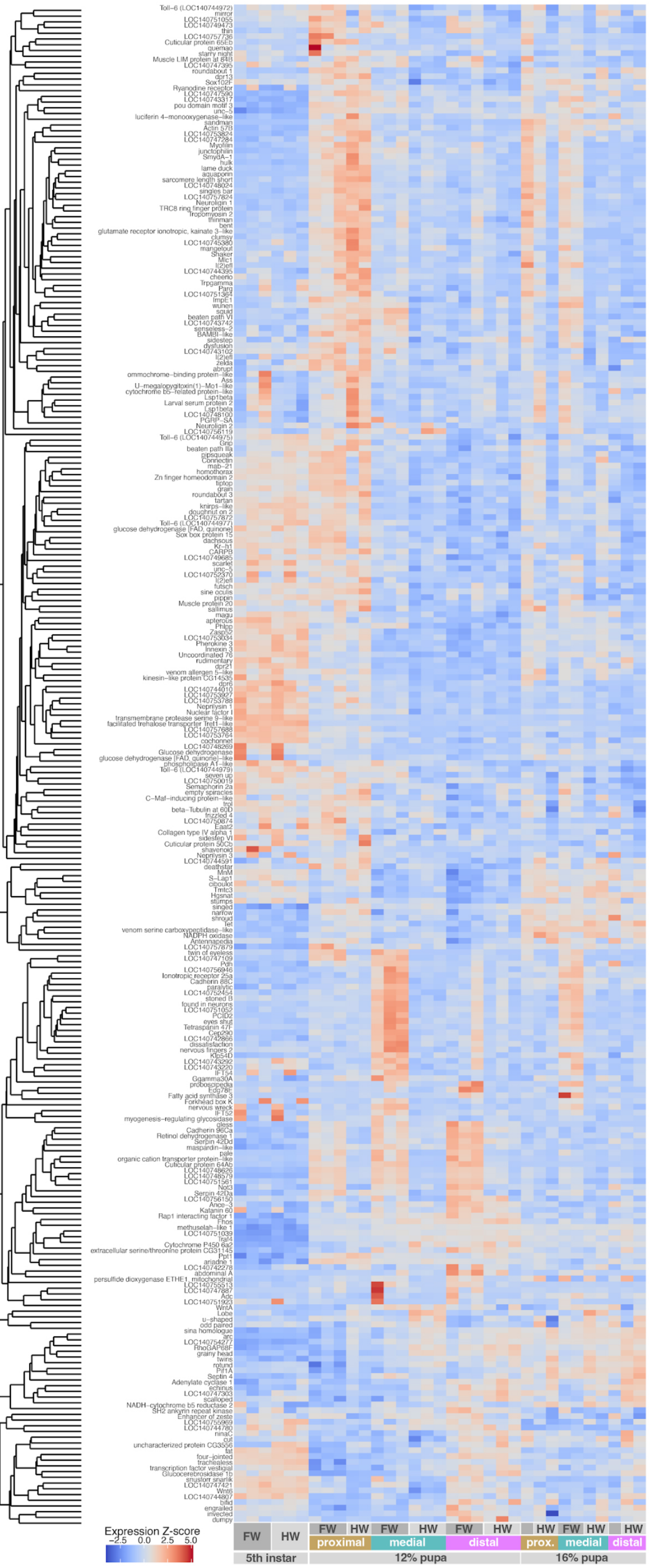

Fig. S2. Heatmap of all 265 P-D axis DEGs. Heatmap of all 265 proximo-distal axis differentially expressed genes, color-coded using a z-score normalization of their expression levels across all samples and time points. A dendrogram reflects the hierarchical clustering of genes by similar expression profiles.

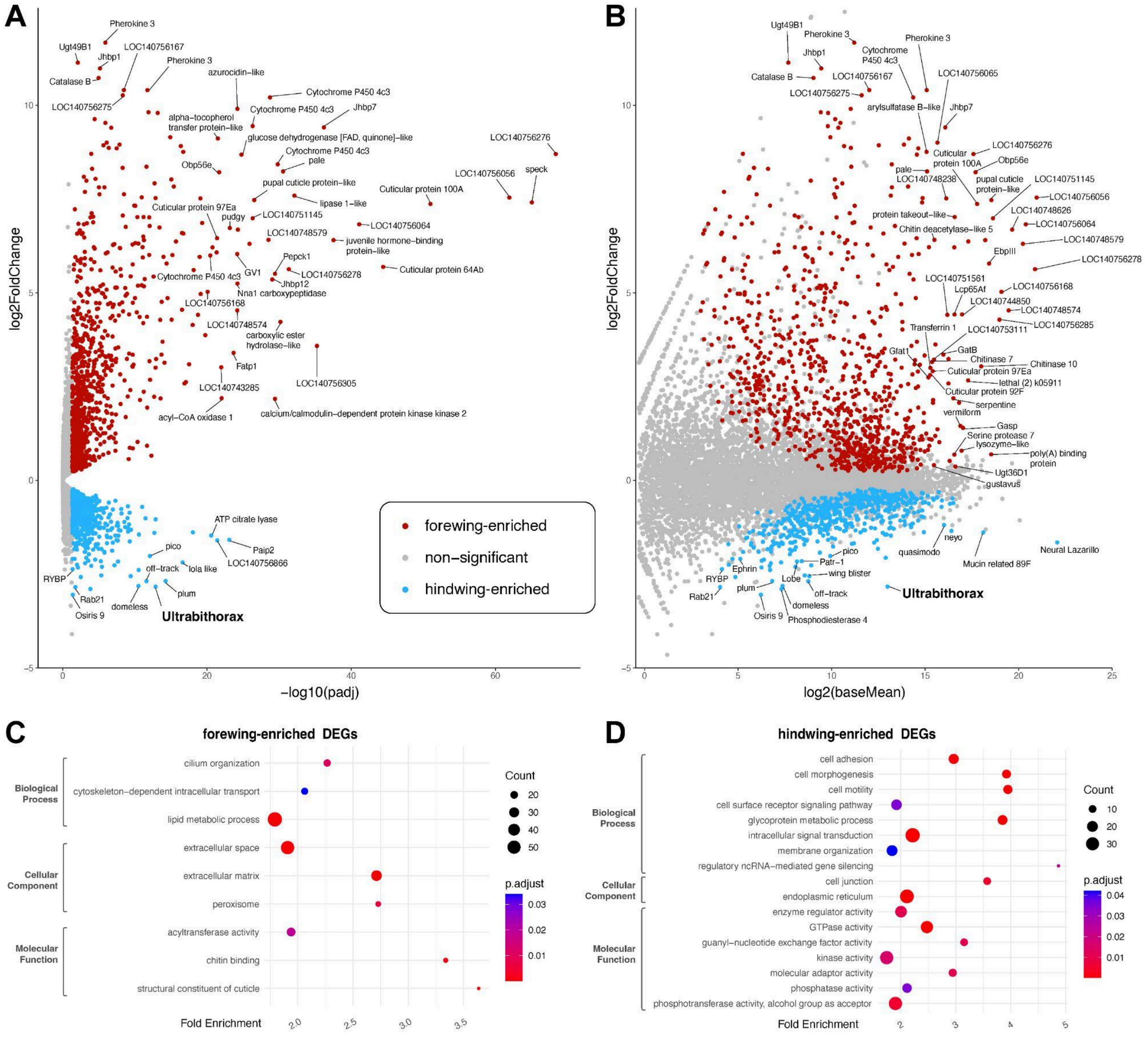

Fig. S3. Significance, expression level, and GO enrichment analyses of differential gene expression between the 12% pupal forewings and hindwings. Transcriptomes appear asymmetric between the forewing and hindwing. This is partly due to the high expression of forewing-specific genes, many of which with annotations associated with cuticle development, suggesting this reflects a role of the forewing in the maturation of cuticle, perhaps relating to the juxtaposition of the forewing and pupal case. **A.** Volcano plot of 12% pupae forewing vs hindwing DEGs. The y-axis is Log2FoldChange Expression and the x-axis is -log10(adjusted p-value). **B.** MA-plot visualization of the same genes. The y-axis is log2FoldChange Expression, and the x-axis is the log2 of average normalized expression (baseMean in DEseq2). **C.** Results of a GO enrichment analysis of forewing-enriched DEGs, using the *slimGO_Drosophila* standardized subset. Enriched categories are clustered by ontological classes (Biological Process, Cellular Component, and Molecular Function). **D.** The same analysis performed for hindwing-enriched DEGs.

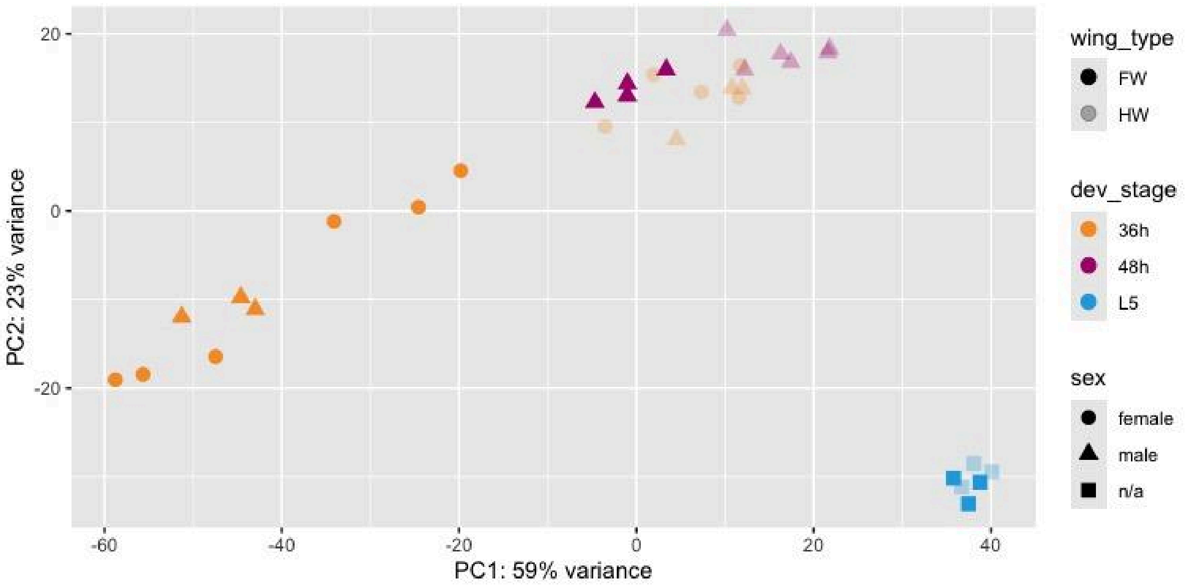

Fig. S4. Principle Component Analysis of gene expression variation among the RNAseq samples. All samples from the analysis, without wing sections collapsed, are shown in this PCA. For each sample, wing type is illustrated (forewing or hindwing), developmental stage (12% [36h], or 16% [48h] pupal development) , and sex. Sex was not determined in the fifth instar larval samples (L5). Among pupal samples, most of the observed variation occurs among forewing samples at the 12% stage, possibly because of the inclusion of cuticle-producing cells (peripodial membrane) at this early stage.

Dataset 1. Manually curated GTF Genome Annotation File

Available for download at
https://journals.biologists.com/bio/article-lookup/doi/10.1242/bio.062297#supplementary-data

Dataset 2. Manually curated Gene Annotation List

Available for download at
https://journals.biologists.com/bio/article-lookup/doi/10.1242/bio.062297#supplementary-data

Biology Open • Supplementary information

Table S1. Table containing the DESeq2 normalized counts for all genes in the genome produced during the forewing vs hindwing (wing-type) analysis

Available for download at
https://journals.biologists.com/bio/article-lookup/doi/10.1242/bio.062297#supplementary-data

Table S2. Table containing the DESeq2 Results from the forewing vs hindwing (wing-type) analysis

Available for download at
https://journals.biologists.com/bio/article-lookup/doi/10.1242/bio.062297#supplementary-data

Table S3. Table containing the DESeq2 normalized counts for all genes in the genome produced during the wing compartment analysis at 12% pupal development

Available for download at
https://journals.biologists.com/bio/article-lookup/doi/10.1242/bio.062297#supplementary-data

Table S4. Table containing the DESeq2 Results from the forewing proximal vs forewing medial compartment analysis at 12% pupal development

Available for download at
https://journals.biologists.com/bio/article-lookup/doi/10.1242/bio.062297#supplementary-data

Table S5. Table containing the DESeq2 Results from the forewing medial vs forewing distal compartment analysis at 12% pupal development

Available for download at
https://journals.biologists.com/bio/article-lookup/doi/10.1242/bio.062297#supplementary-data

Table S6. Table containing the DESeq2 Results from the forewing proximal vs forewing distal compartment analysis at 12% pupal development

Available for download at
https://journals.biologists.com/bio/article-lookup/doi/10.1242/bio.062297#supplementary-data

Biology Open • Supplementary information

Table S7. Table containing the DESeq2 Results from the hindwing proximal vs hindwing medial compartment analysis at 12% pupal development

Available for download at
https://journals.biologists.com/bio/article-lookup/doi/10.1242/bio.062297#supplementary-data

Table S8. Table containing the DESeq2 Results from the hindwing medial vs hindwing distal compartment analysis at 12% pupal development

Available for download at
https://journals.biologists.com/bio/article-lookup/doi/10.1242/bio.062297#supplementary-data

Table S9. Table containing the DESeq2 Results from the hindwing proximal vs hindwing distal compartment analysis at 12% pupal development

Available for download at
https://journals.biologists.com/bio/article-lookup/doi/10.1242/bio.062297#supplementary-data

Table S10. Table containing the DESeq2 normalized counts for all genes in the genome per wing compartment analysis for all developmental timepoints

Available for download at
https://journals.biologists.com/bio/article-lookup/doi/10.1242/bio.062297#supplementary-data

Table S11. Table containing the data plotted in the full heatmap in Fig. S2

Available for download at
https://journals.biologists.com/bio/article-lookup/doi/10.1242/bio.062297#supplementary-data

Biology Open • Supplementary information
